# Effects of increasing standard Ileal digestible lysine and metabolizable energy levels in lactation diets fed to young and mature sows

**DOI:** 10.1093/jas/skae247

**Published:** 2024-08-24

**Authors:** Jorge Estrada, Jeremy G Perez, Kelsey L Kyle, Eric Parr, Michael W Welch, Danielle C Johnson, Casey Neill, Dustin D Boler

**Affiliations:** Carthage Veterinary Service Ltd, Carthage, IL 62321, USA; Carthage Veterinary Service Ltd, Carthage, IL 62321, USA; Carthage Veterinary Service Ltd, Carthage, IL 62321, USA; Carthage Veterinary Service Ltd, Carthage, IL 62321, USA; Carthage Veterinary Service Ltd, Carthage, IL 62321, USA; Carthage Veterinary Service Ltd, Carthage, IL 62321, USA; Carthage Veterinary Service Ltd, Carthage, IL 62321, USA; Carthage Veterinary Service Ltd, Carthage, IL 62321, USA

**Keywords:** breeding performance, energy, farrowing performance, lactation, lysine, pig

## Abstract

The objective was to evaluate increasing standard ileal digestible (**SID**) lysine (Lys) and metabolizable energy (**ME**) above recommended inclusion levels fed to young (parity 1 to 2) and mature (parity 3 to 5) lactating sows. A total of 741 sows (371 parity 1 to 2, 370 parity 3 to 5) were fed in a 2 × 2 factorial arrangement of treatments in a randomized complete block design. Factors included SID Lys (0.85% vs. 1.11%) and ME (3.18 Mcal/kg vs. 3.33 Mcal/kg). Diets were formulated to deliver 62 g of SID Lys at 0.85% inclusion and 23.2 Mcal of energy to sows that consume 7.30 kg of complete feed per day. Overall, sows consumed 61 g/d SID Lys and 22.6 Mcal/kg, but because of nutrient intake differences between young and mature sows, outcome variables were analyzed separately and not statistically compared. There were no differences for either SID Lys or ME treatment in lactation feed intake (*P* ≥ 0.27) or change in body weight (*P* ≥ 0.19) for young sows. Young sows fed 3.33 Mcal/kg ME had 0.31 fewer post-cross foster mortalities (*P* < 0.01) resulting in weaned litters that were 4.06 kg heavier (*P* < 0.01) than litters from young sows fed 3.18 Mcal/kg ME. Young sows fed 1.11% SID Lys and 3.33 Mcal/kg ME had the fewest (*P* ≤ 0.03) sows bred by day 7 compared to all other treatments. There was an interaction (*P* = 0.03) for wean-to-estrus interval for young sows were young sows fed 1.11% SID Lys and 3.33 Mcal/kg ME were at least 3.27 d longer than young sows fed 0.85% SID Lys and 3.33 Mcal/kg ME or 1.11% SID Lys and 3.18 Mcal/kg ME. Young sows fed 3.33 Mcal/kg ME had a nearly 7% improvement in lactation G:F (0.40 vs. 0.43; *P* < 0.01) compared to young sows fed 3.18 Mcal/kg ME. The percentage of mature sows fed 1.11% SID Lys bred by day 7 was 7.0 percentage units less (*P* = 0.06) when fed 3.33 Mcal/kg ME compared to 3.18 Mcal/kg ME. Feed efficiency of mature sows fed 1.11% SID Lys (0.38) was 7.89% greater (*P* < 0.01) than mature sows fed 0.85% SID Lys (0.35). Increasing either SID Lys or ME energy to young and mature sows had little influence on sow or pig performance, but simultaneously increasing both SID Lys and ME negatively impacted subsequent breeding performance.

## Teaser

This study investigated the effects of simultaneously feeding greater than recommended levels of lysine and energy to lactating sows. The research highlights the importance of considering the relationship between levels of SID lysine and metabolizable energy on sow farrowing performance, pig performance, and subsequent breeding performance in young and mature sows.

## Introduction

Modern lactating sows require at least 60 g of standard ileal digestible (**SID**) lysine (Lys) per day to optimize litter growth (Tokach et al., 2019) and at least 21 Mcal metabolizable energy (**ME**)/kg for body composition maintenance and milk production. ME needs were based on a model that accounts for a sows body weight and the total number of pigs born in a litter. Total pigs born per litter have increased from less than 10 in 2008 (USDA, 2010) to 15.4 in 2024 (MetaFarms, 2024), meaning ME requirements of modern lactating sows have also increased since the release of the most recent nutrient recommendations. Formulating lactation diets to deliver adequate nutrients is also important for sows to ensure milk protein output is sufficient for the success of the litter (Strathe et al., 2017) and to reduce muscle protein mobilization in lactating sows (Gourley et al., 2017; Pedersen et al., 2019). Insufficient lactation daily feed intakes lead to poor sow body condition and reproductive failure (Koketsu et al., 1996), an increase in preweaning mortality, and decreased pig weaning weights (Koketsu et al., 1996; Prunier et al., 1997; Sulabo et al., 2010).

It is well established that increasing Lys during lactation reduces body weight loss, presumably by decreasing mobilization of muscle protein (Xue et al., 2012; Shi et al., 2015; Gourley et al., 2017). Increasing energy by 4.5% (3.06 vs. 3.20 Megacalories (Mcal)/kg of ME) reduced sow weight loss and increased litter growth rate during lactation (Xue et al., 2012). However, diets with even greater energy concentration (3.30 to 3.40 Mcal/kg ME) reduced feed intake (Xue et al., 2012) and did not increase overall energy consumption.

Estrada et al. (2024) reported variation in SID Lys intake can be as high as 67% and ME intake variation as high as 68%. Most nutrient intake variation was related to parity differences. Estrada et al. (2024) reported younger sows had less feed intake than mature sows and a greater percentage of young sows consumed less than 60 g of Lys per day. Dourmad et al. (2008) proposed that mobilization of protein and energy may be interdependent, therefore, the interaction between amino acids (**AA**) and energy requirements must be considered. Providing sows with calorically dense diets is important for maintaining optimal weight and body condition. However, sows fed high-energy diets might consume fewer total grams of Lys, resulting in reduced litter performance. Therefore, the objective was to evaluate the synergistic effects of feeding young and mature lactating sows diets that exceed formulated recommendations for both SID lysine and ME.

## Materials and Methods

All experimental procedures were reviewed and approved by the Carthage Veterinary Services IACUC committee (protocol #2023-16).

### Animals and housing

A total of 741 (371 parity 1 to 2, 370 parity 3 to 5, 114 parity 6+) sows (PIC 1050 Camborough) were fed in the summer of 2023 at a commercial sow farm near Carthage, IL. No sows that were parity 6 or older were included in the data set. Parity 1 and 2 sows were considered young sows and parity 3 to 5 were considered mature sows. Sows were moved from the gestation pens into the farrowing room at a target of 112 d of gestation. Upon entering the farrowing room, sows were weighed, measured with a caliper (scale 1 to 20; <8 considered thin, >11 considered over-conditioned; Knauer and Baitinger. 2015) and scanned for backfat depth using a Renco fat probe (Renco Corporation, Minneapolis, MN) at the area of the last rib targeting 65 mm from the midline. Each farrowing stall (1.5 m × 2.1 m total, with 0.6 m × 2.1 m for sows) had a nipple waterer and a dry box feeder for the sow, and a heat lamp for the pigs.

### Diets, feeding, and experimental design

All diets were formulated (Table 1) to meet or exceed the current National Research Council’s (2012) guidelines and PIC’s recommendations for lactating sows. Diets were manufactured at a commercial feed mill in Carthage, IL. Diets were formulated to deliver 62 g of SID Lys at 0.85% inclusion and 23.2 Mcal of ME to sows that consume 7.30 kg of complete feed per day (Tables 1 and 2). Sows were fed a standard gestation diet the day they were moved into the farrowing room and started on their respective research diet the following day. Sows were then fed experimental diets until weaning.

**Table 1.** Lactation diet formulation

	Treatment			
	0.85% SID Lys	0.85% SID Lys	1.11% SID Lys	1.11% SID Lys
Ingredient, %	3.18 Mcal/kg	3.33 Mcal/kg	3.18 Mcal/kg	3.33 Mcal/kg
Corn	71.02	67.89	59.67	56.62
Soybean meal	15.05	15.50	26.50	26.70
Corn oil	0.75	3.55	0.75	3.60
DDGS	9.00	9.00	9.00	9.00
Calcium carbonate	1.20	1.19	1.23	1.22
Monocal 21% P	1.03	1.04	0.87	0.88
Salt	0.51	0.51	0.50	0.50
l-Lysine HCl 78%	0.38	0.38	0.38	0.38
l-Threonine 99%	0.12	0.12	0.14	0.14
dl-Methionine 99%	0.02	0.02	0.06	0.07
l-Tryptophan 98.5%	0.04	0.03	0.02	0.02
Vitamin/trace mineral premix	0.25	0.25	0.25	0.25
Choline chloride 60%	0.10	0.10	0.10	0.10
Laxative (Mg K SO_4_)	0.35	0.35	0.35	0.35
Flow agent—clay	0.07	0.07	0.07	0.07
Microgrits orange color	0.10	0.00	0.00	0.00
Iron oxide-Black color	0.00	0.00	0.10	0.00
Iron oxide-Red color	0.00	0.00	0.00	0.10
Total	100.00	100.00	100.00	100.00

**Table 2.** Lactation nutrient composition of experimental diets (as-fed basis)

	Treatment							
	0.85% SID Lysine		0.85% SID Lysine		1.11% SID Lysine		1.11% SID Lysine	
	3.18 Mcal/kg		3.33 Mcal/kg		3.18 Mcal/kg		3.33 Mcal/kg	
Composition^1^	Calc.	Analy.	Calc.	Analy.	Calc.	Analy.	Calc.	Analy.
Metabolizable energy, kcal/kg	3,182	—	3,334	—	3,183	—	3,334	—
Moisture^2^	13.98	13.69	13.56	13.72	13.71	13.37	13.28	13.10
Crude protein^2^, %	15.43	15.23	15.39	15.53	19.83	20.11	19.70	19.55
Crude fat^2^, %	4.18	3.75	6.74	6.03	3.99	3.56	6.60	6.10
Ash^2^, %	5.14	4.81	5.12	4.76	5.62	5.32	5.50	5.36
NDF^2^, %	9.59	8.60	9.38	8.05	9.60	8.56	9.37	8.00
Analyzed Ca^2^, %	0.73	0.70	0.72	0.74	0.75	0.74	0.74	0.80
Phosphorus^2^, %	0.54	0.55	0.54	0.57	0.55	0.57	0.55	0.59
Ca:P	1.35	1.28	1.35	1.30	1.35	1.30	1.35	1.36
STTD phosphorus, %	0.46	—	0.46	—	0.46	—	0.46	—
Phytase, units/kg	750	—	750	—	750	—	750	—
Zinc^2^, ppm	153.26	174.08	152.98	172.23	157.80	167.50	157.39	211.80
Iron^2^, ppm	223.75	264.30	223.73	282.90	897.86	346.20	741.36	410.80
Manganese^2^, ppm	61.47	88.38	61.37	89.54	63.60	86.81	63.45	98.66
Copper^2^, ppm	38.81	26.09	38.79	32.95	40.24	28.62	40.18	32.96
Sodium^2^, %	0.23	0.24	0.23	0.21	0.23	0.21	0.23	0.23
Lysine^3^, %	0.97	0.91	0.97	1.00	1.26	1.26	1.26	1.25
Isoleucine^3^, %	0.58	0.55	0.58	0.59	0.78	0.82	0.78	0.79
Leucine^3^, %	1.44	1.36	1.43	1.45	1.73	1.76	1.71	1.73
Met + Cys^3^, %	0.55	0.50	0.54	0.53	0.69	0.65	0.69	0.64
Threonine^3^, %	0.65	0.65	0.65	0.67	0.84	0.86	0.84	0.82
Tryptophan^3^, %	0.19	0.20	0.18	0.19	0.24	0.23	0.24	0.24
Valine^3^, %	0.69	0.65	0.69	0.69	0.89	0.89	0.88	0.88
SID Lysine, %	0.85	—	0.85	—	1.11	—	1.11	—
SID Lys:Cal ME ratio	2.67	—	2.56	—	3.49	—	3.33	—
Total AA:total Lys (ratio)								
Met:Lys	0.29	0.29	0.29	0.27	0.30	0.28	0.30	0.27
Met + Cys:Lys	0.56	0.55	0.56	0.53	0.55	0.51	0.55	0.51
Trp:Lys	0.19	0.22	0.19	0.19	0.19	0.18	0.19	0.19
Thr:Lys	0.67	0.71	0.67	0.67	0.67	0.68	0.66	0.66
Leu:Lys	1.48	1.49	1.47	1.44	1.37	1.39	1.36	1.38
Iso:Lys	0.60	0.60	0.60	0.59	0.62	0.65	0.62	0.63
Val:Lys	0.71	0.71	0.70	0.69	0.70	0.71	0.70	0.71

^1^Diets were in meal form and manufactured at the NSI feed mill.

^2^Analyses were carried out by Midwest Labs using wet chemistry.

^3^Analyses were carried out by Ajinomoto Animal Nutrition North America, Inc. (Eddyville, IA).

**Figure 1.**
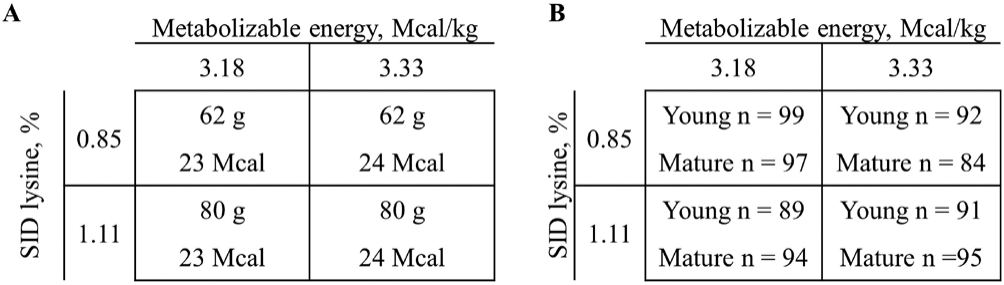
(A) Targeted daily SID lysine and metabolizable energy (ME) intake per treatment. Nutrient targets were based on projected sow daily feed intake of 7.30 kg/d (Estrada et al., 2024, doi:10.1093/jas/skae093). (B) Number (*n*) of young and mature sows per treatment combination to complete the trial.

Sows were initially blocked by parity (1, 2, 3, and 4+) and randomly allotted to 1 of 4 treatments with factors being SID Lys (0.85% vs. 1.11%) and ME (3.18 Mcal/kg vs. 3.33 Mcal/kg, Figure 1A). Overall, 196 sows (including 99 young and 97 mature; Figure 1B) were fed treatment 1 (0.85% SID Lys, 3.18 Mcal/kg ME), 176 (92 young and 84 mature) fed treatment 2 (0.85% SID Lys, 3.33 Mcal/kg ME), 183 (89 young and 94 mature) fed treatment 3 (1.11% SID Lys, 3.18 Mcal/kg ME), and 186 (91 young and 95 mature) fed treatment 4 (1.11% SID Lys, 3.33 Mcal/kg ME). Prior to farrowing, all sows were fed 2.27 kg of complete feed per day using a calibrated volumetric scoop, fed in 2 equal-sized meals in the morning and afternoon. After farrowing, all sows were allowed ad libitum access to lactation feed based on specific treatment assignments. Feed added to the feeders was recorded daily and feed remaining in the feeders on day of farrowing and after weaning was measured to calculate individual feed disappearance from loading until farrowing, farrowing until weaning, and overall feed intake.

Research diets were manufactured at a commercial toll mill. Reasonable variation in formulated and analyzed nutrient values should be expected. Even so, formulated nutrient intake targets had less than 5% error rate for all 4 treatment combinations. Further, young sows consumed less than formulated nutrient targets and mature sows consumed more than formulated nutrient targets. These outcomes justify segregating the data into young and mature sows and provide credibility to the accuracy of the manufactured diets. A weekly sample of the lactation diet was collected and sent to a commercial laboratory (Midwest Laboratories, Omaha, NE) and analyzed in duplicate for crude protein (AOAC 990.03, 2006), crude fat (AOAC 2003.05, 2007), neutral detergent fiber (Ankom Technology/AOAC 2001.11, 2005), ash (AOAC 942.05, 2012), calcium, phosphorous, sodium, iron, manganese, copper, and zinc (AOAC 985.01, 1996). A composite sample of each diet was sent to Ajinomoto Laboratory (Eddyville, IA) for amino acid (AA) analysis (994.12, AOAC 2012).

### Farrowing and data collection

Sows were monitored every 30 min between 0645 and 1500 hours to record the first pig born. Sows that farrowed overnight were checked the following morning. Sows that had not yet farrowed by day 116 of gestation were induced with PGF2α (2c.c., Lutalyse, Zoetis Inc., Kalamazoo, MI). Within 24 h of farrowing, numbers of total pigs born, total live pigs born, stillbirths, and mummies were recorded for each litter. Live pigs were weighed as a group to record litter birth weight. Weights of stillbirth pigs and mummies were weighed and added to the litter birth weight to calculate a litter average pig birth weight. Empty sow weight after farrowing was estimated by subtracting conceptus weight from pre-farrow adjusted weight. Conceptus weight was estimated using the equation: 0.137 + 1.329 × total pigs born × litter average pig birth weight (Estrada et al., 2024). Pre-farrow adjusted weight was estimated as total pigs born × 0.039 × days until farrowing from time of weighing + pre-farrow weight (Estrada et al., 2024). Equations were adapted from historical estimations of sow weight and conceptus weight (Mallmann et al., 2018; Thomas et al., 2018).

Adequate colostrum intake was ensured for all pigs by following the Carthage System’s standard operating procedure for day 1 pig care. During and shortly after parturition pigs were placed at the underline of the sow to encourage nursing. Pigs that were suspected of not nursing were placed under a heat lamp or in a warming box if chilled. A split-suckling approach was used where pigs that had been observed nursing were moved to a warming box to ensure all pigs had access to colostrum. Those that were not observed nursing were marked and placed at the underline of the sow. Pigs were not away from the sow for more than 45 min. All pigs were required to be observed nursing their biological mother before it could be moved to a new sow.

Pigs were cross fostered within treatment to equalize litter size within 48 h of farrowing. The number of functional teats was counted for each sow and compared to the number of pigs the sow farrowed. Pigs to be cross fostered were weighed and carried by hand from the crate of the birth sow to the crate of the recipient sow to ensure no sow was responsible for more pigs than she had available functional teats. All movements were recorded, including the date of cross fostering and identification of the birth and recipient sows. The number of pigs was verified and recorded upon completion of cross fostering. Litter starting weight was calculated by adjusting the litter birth weight for weight of cross fostered pigs and the weight of the pigs that died prior to cross fostering. The weight of the pigs that died prior to cross-fostering was subtracted from the litter birth weight to get a starting weight used to calculate litter average daily gain. Litter gain was calculated by subtracting the litter starting weight from the litter weaning weight. Litter ADG was calculated by dividing litter gain by the number of days that elapsed from the starting weight and weaning weight. The date, weight, and reason for mortality were recorded for all pigs. Pigs that were removed due to weight loss or injury were categorized as a falloff and were weighed and moved to a sow that was not enrolled in the trial.

Individual pig weaning weights were recorded and used to calculate litter weaning weight. Litter gain was calculated by subtracting litter starting weight from litter weaning weight. At weaning, sows were weighed, measured with a caliper (Knauer and Baitinger. 2015), and scanned for backfat depth. Sow weight change was calculated by subtracting the calculated sow weight after farrowing from the sow weight at weaning. Lactation feed efficiency was calculated by subtracting litter starting weight from litter weaning weight and dividing by total sow lactation feed intake. Sows were then moved to gestation crates and monitored after weaning until they showed signs of estrus.

### Statistical analysis

Analyses were conducted using the Mixed procedure of SAS version 9.4 (SAS, Cary, NC). Sows were randomly assigned to treatment within parity group and blocked by replication (one sow per treatment combination per replicate) within wave (groups of approximately 250 sows per wave. Data were analyzed as a 2 × 2 factorial arrangement of treatments coded by lysine level (0.85% vs. 1.11%) and ME level (3.18 Mcal/kg vs. 3.33 Mcal/kg) and their interactions as fixed factors. Block was included as a random variable. Uncertainty of the estimates of least squares means was expressed as the maximum standard error among the 4 treatment combinations. Least squares mean differences for main effects and interactions were considered statistically different from 0 at *P* < 0.05 and tendencies at *P* < 0.10. Means separation was determined using the PDIFF option in the Mixed procedure of SAS without an adjustment for multiple comparisons.

The percentage of sows bred by day 4 and day 7 after weaning was calculated using the Glimmix procedure in SAS (SAS Inst. Inc.). A binomial distribution with a logit link function was used to calculate least squares means of both outcome variables. A Kenward–Rogers degrees of freedom approximation was used to compute the denominator degrees of freedom due to differences in observations across treatments. The model included the fixed effect of SID lysine, ME, and the interaction between lysine and energy. Means separation was determined using the PDIFF option in the Glimmix procedure and mean output was provided using the ilink option.

## Results

The average total number of pigs born was 16.83 (Table 3), which was similar to the national average of 15.4 (MetaFarms, 2024). Sows in this trial weaned between 7 and 17 pigs per litter (Table 3). Average pigs weaned per sow was 13.15 (Table 3) which is representative of the U.S. national average (MetaFarms, 2024).The population of sows in this trial also had an average litter wean weight of 68.76 kg and an average litter gain of 49.96 kg (Table 3). Therefore, these sows are representative of U.S. industry standards.

**Table 3.** Population summary statistics of sow and pig performance

Item	Observations	Mean	Median	SD	Minimum	Maximum	CV
Backfat at entry into farrowing, mm	633	10.97	11.00	2.78	5.00	23.00	25.35
Backfat at weaning, mm	632	9.94	10.00	2.21	4.00	19.00	22.20
Sow weight into farrowing, kg	637	228.41	227.91	25.53	165.71	319.62	11.18
Sow weight at weaning, kg	635	203.57	203.39	28.05	138.02	286.02	13.78
Estimated sow weight after farrowing^1^, kg	637	202.10	200.89	27.45	136.81	291.21	13.58
Lactation daily feed intake, kg	634	6.99	7.03	1.11	3.92	9.88	15.86
Total lactation feed intake, kg	634	131.19	130.41	23.17	69.46	207.48	17.66
Total born, *n*	721	16.83	17.00	3.36	6.00	27.00	19.96
Born alive, *n*	721	15.09	15.00	3.08	4.00	24.00	20.43
Stillborn, *n*	721	1.36	1.00	1.60	0.00	15.00	117.50
Mummies, *n*	721	0.38	0.00	0.79	0.00	7.00	208.00
Starting litter inventory, *n*	639	14.60	15.00	1.29	10.00	17.00	8.83
Pigs weaned, *n*	637	13.15	13.00	1.79	7.00	17.00	13.62
Litter birth weight, kg	718	20.95	21.13	3.82	5.9	33.10	18.21
Litter starting weight^2^, kg	638	21.08	21.27	3.94	10.44	32.55	18.67
Litter wean weight^3^, kg	637	68.76	69.74	11.88	31.14	100.64	17.27
Litter gain^4^, kg	636	49.96	49.97	9.19	24.15	73.86	18.39
Lactation G:F^5^	633	0.37	0.37	0.08	0.11	0.66	22.74
Average birth weight of pigs, kg	717	1.41	1.39	0.21	0.83	2.25	15.20
Average starting weight of pigs^2^, kg	636	1.45	1.46	0.25	0.77	2.68	17.51
Average wean weight^3^, kg	637	5.24	5.20	0.67	3.50	7.25	12.81
Average daily pig gain, kg/d	636	0.21	0.21	0.03	0.12	0.29	14.50

^1^Estimated sow weight into farrowing = pre-farrow adjusted weight—conceptus weight. Pre-farrow adjusted weight = total born × 0.039 × days until farrowing from time of weighing + pre-farrowing weight. Sow weight into farrowing was the weight of the sow when placed in the farrowing crate at approximately day 112 of gestation. Conceptus weight = 0.137 + 1.329 × total pigs born × average pig birth weight (Estrada et al., 2024, doi:10.1093/jas/skae093).

^2^Litter starting weight = weight after 48 h cross foster period.

^3^Pig weaning weights were obtained an average of 1.35 d prior to weaning, therefore, lactation length is from date of farrowing until date that weaning weights were obtained.

^4^Litter gain = litter weaning weight − litter starting weight.

^5^Lactation gain-to-feed (G:F) was calculated as (litter weaning weight − litter starting weight) ÷ total sow lactation feed intake.

### Young sow performance

**Figure 2.**
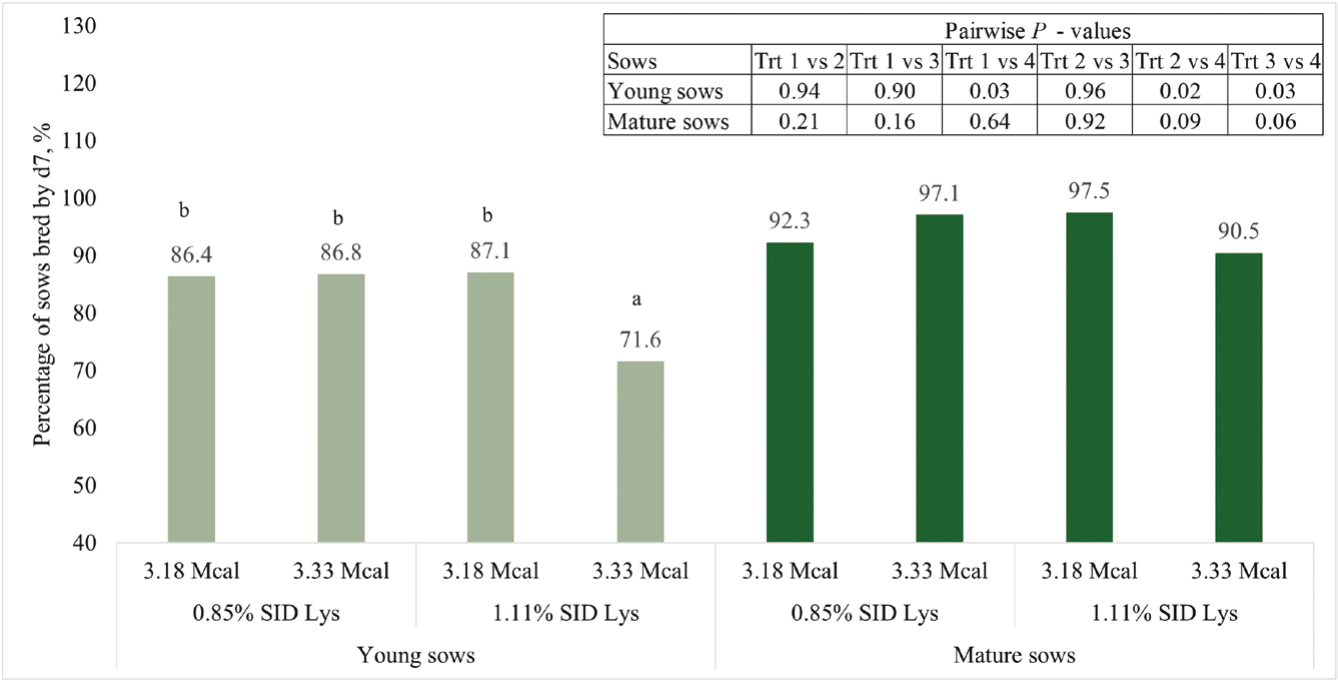
Percentage of sows bred by day 7 after weaning. Treatment means within sow group (young vs. mature) in the bar graph that do not share a superscript differ (*P* < 0.05).

**Figure 3.**
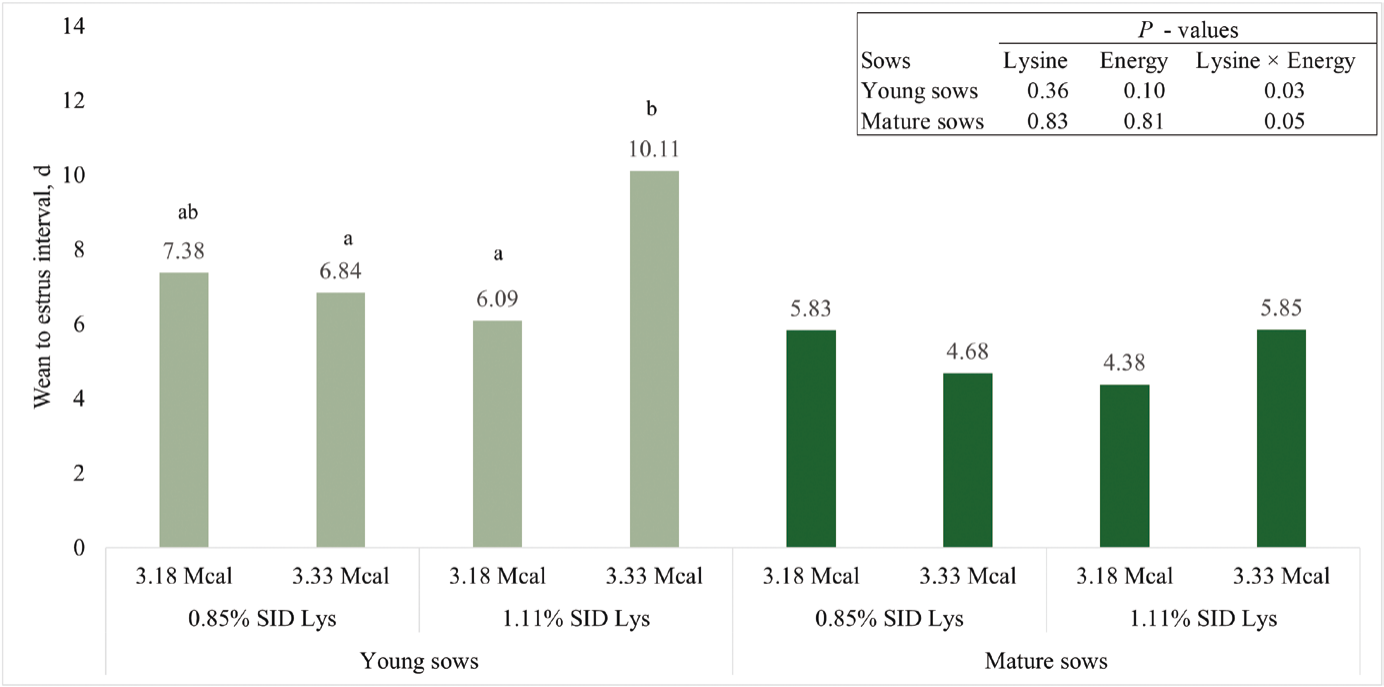
Number of days after weaning before sows exhibited signs of estrus. Treatment means within sow group (young vs. mature) in the bar graph that do not share a superscript differ (*P* < 0.05).

There was no interaction (*P* = 0.35) among young sows for caliper at entry into farrowing (Table 4). There was an interaction (*P* = 0.03) between SID Lys and ME for caliper measurement at weaning (Table 4). Young sows fed 1.11% SID Lys and 3.18 Mcal/kg ME had caliper scores that were 0.78 units greater (*P* < 0.01) than young sows fed 1.11% SID Lys and 3.33 Mcal/kg ME and 0.64 units greater (*P* = 0.03) than young sows fed 0.85% SID Lys and 3.18 Mcal/kg ME (Table 4). There was no interaction (*P* = 0.97) for body condition score at farrowing (Table 4). There was an interaction (*P* = 0.03) for body condition score at weaning (Table 4). Young sows fed 1.11% SID Lys and 3.33 Mcal/kg ME had body condition scores that were at least 0.13 units less (*P* ≤ 0.05) than the other 3 treatments (Table 4). There were no interactions for backfat measurement at entry into farrowing (*P* = 0.84) or at weaning (*P* = 0.55, Table 4). However, backfat measurements at weaning were 0.48 mm greater (*P* = 0.04) for young sows fed 0.85% SID Lys compared to 1.11% SID Lys. There were no interactions for backfat change during lactation (*P* = 0.79), sow weight into farrowing (*P* = 0.93), estimated sow weight after farrowing (*P* = 0.89), conceptus weight (*P* = 0.33), sow weight at weaning (*P* = 0.80), sow weight change during lactation (*P* = 0.92), percent of sows that were culled (*P* = 0.51), and percent of sows that were bred be day 4 after weaning (*P* = 0.33, Table 4). There was an interaction for percent of young sows bred by 7 d after weaning (Figure 2). Young sows fed 1.11% SID Lys and 3.33 Mcal/kg ME had the fewest sows bred *(P *≤ 0.03) by day 7 compared to all other treatments. There was also an interaction (*P* = 0.03) for wean-to-estrus interval of young sows (Figure 3). Young sows fed 1.11% SID Lys had an average wean-to-estrus interval 4.02 d longer (*P *< 0.01) when fed 3.33 Mcal/kg ME compared to sows fed 1.11% SID Lys and 3.18 Mcal/kg ME (Figure 3), but there were no differences in wean-to-estrus interval between sows fed 0.85% SID Lys regardless of ME inclusion. There were also no differences among young sows fed 0.85% SID Lys regardless of ME and young sows fed 1.11% SID Lys and 3.33 Mcal/kg ME (Figure 3).

**Table 4.** Interactive least squares means of young sow (parity 1 and 2) weight and body composition^1^

	Treatment (% SID lysine and Mcal/kg, metabolizable energy)					*P* values		
	0.85	0.85	1.11	1.11				Lysine ×
Item	3.18	3.33	3.18	3.33	SEM	Lysine	Energy	Energy
Sows, *n*	99	92	89	91				
Parity	1.51	1.51	1.52	1.53	0.53	0.79	0.88	0.96
Caliper at entry into farrowing	8.87	8.87	9.04	8.61	0.28	0.84	0.35	0.35
Caliper at weaning	6.89^a^	7.02^ab^	7.53^b^	6.75^a^	0.21	0.37	0.12	0.03
Body condition score (BCS) into farrowing	2.99	2.92	2.93	2.87	0.07	0.31	0.23	0.97
Body condition score (BCS) at weaning	2.60^b^	2.55^b^	2.67^b^	2.42^a^	0.06	0.51	<0.01	0.03
Backfat at entry into farrowing, mm	10.95	10.69	10.54	10.16	0.43	0.13	0.30	0.84
Backfat at weaning, mm	9.98	9.83	9.63	9.21	0.31	0.04	0.23	0.55
Backfat change during lactation, mm	−1.11	−0.97	−0.99	−0.99	0.38	0.87	0.81	0.79
Sow weight into farrowing, kg	211.95	211.01	212.95	211.68	2.06	0.66	0.56	0.93
Estimated sow weight after farrowing^2^, kg	186.91	186.04	187.80	187.41	1.79	0.52	0.72	0.89
Conceptus weight^3^, kg	28.39	28.50	28.73	27.66	0.62	0.68	0.43	0.33
Sow weight at weaning, kg	186.50	183.98	186.44	182.79	2.28	0.78	0.17	0.80
Sow weight change during lactation^4^, kg	−2.21	−5.56	−2.10	−7.00	3.22	0.83	0.19	0.81
Sow weight change during lactation, %	−1.00	−2.99	−1.34	−3.66	1.73	0.76	0.20	0.92
Culled sows, %	15.15	15.22	14.61	19.78	3.92	0.60	0.50	0.51
Sows bred by day 4 after weaning, %	58.02	59.21	61.43	51.35	5.93	0.70	0.44	0.33

^1^A total of 371 parity 1 and 2 sows were used from farrowing until weaning. Sows were weighed, blocked by parity and randomly assigned to 1 of 4 diets with factors being SID lysine (0.85% or 1.11%) and metabolizable energy (3.18 Mcal/kg or 3.33 Mcal/kg). Movement from gestation to farrowing occurred at a target of 112 d of gestation. Weaning occurred at days 14 to 24 of lactation.

^2^Estimated sow weight after farrowing = Adjusted weight before farrowing − conceptus weight. Adjusted weight before farrowing = total piglets born × 0.039 × days until farrowing from time of last weighing + weight into farrowing (Estrada et al., 2024, doi: 10.1093/jas/skae093). Sow weight into farrowing was the weight of the sow when moved into the farrowing crate at approximately 112 d of gestation.

^3^Conceptus weight = 0.137 + 1.329 × total piglets born × average piglet birth weight (Estrada et al., 2024, doi: 10.1093/jas/skae093).

^4^Sow weight change = weight at weaning − estimated sow weight after farrowing.

### Young sow feed consumption

There were no interactions between SID Lys and ME among young sows for daily g/d SID Lys intake (*P* = 0.96), Mcal/d daily ME intake (*P* = 0.91), loading to weaning feed intake (*P* = 0.66), lactation total intake (*P* = 0.68), lactation daily feed intake (*P* = 0.89), or lactation gain-to-feed ratio (*P* = 0.78, Table 5).

**Table 5.** Interactive least squares means of total feed intake and average daily feed intake of young sows (parity 1 and 2) during lactation^1^

	Treatment (% SID lysine and Mcal/kg, metabolizable energy)					*P* values		
	0.85	0.85	1.11	1.11				Lysine ×
Item	3.18	3.33	3.18	3.33	SEM	Lysine	Energy	Energy
Sows, *n*	99	92	89	91				
Parity	1.51	1.51	1.52	1.53	0.53	0.79	0.88	0.96
Daily SID lysine intake, g/d	56.15	55.33	71.11	70.40	1.12	<0.01	0.49	0.96
Daily metabolizable energy intake, Mcal/d	21.14	21.81	20.60	21.35	0.42	0.17	0.05	0.91
Loading to weaning total intake^2^, kg	131.63	128.91	127.77	127.12	2.97	0.22	0.47	0.66
Loading to weaning ADFI^2^, kg	5.78	5.68	5.65	5.61	0.10	0.32	0.48	0.74
Lactation total intake, kg	121.97	119.46	118.45	117.86	3.02	0.27	0.50	0.68
Lactation ADFI, kg	6.65	6.55	6.48	6.41	0.13	0.17	0.47	0.89
Lactation G:F^3^	0.40	0.42	0.41	0.44	0.01	0.11	<0.01	0.78

^1^A total of 371 parity 1 and 2 sows were used from farrowing until weaning. Sows were weighed, blocked by parity and randomly assigned to 1 of 4 diets with factors being SID lysine (0.85% or 1.11%) and metabolizable energy (3.18 Mcal/kg or 3.33 Mcal/kg). Movement from gestation to farrowing occurred at a target of 112 d of gestation. Weaning occurred at days 14 to 24 of lactation.

^2^Time of movement from gestation to farrowing room through weaning of the piglets.

^3^Lactation G:F = (litter weaning weight—litter starting weight) ÷ sow total lactation feed intake.

Young sows fed 1.11% SID Lys (70.75 g/d) consumed 15 g SID Lys more (*P* < 0.0001) per day than young sows fed 0.85% SID Lys (55.74 g/d, Table 5). Young sows fed 3.33 Mcal/kg ME (21.58 Mcal) tended to consume 3.28% (0.71 Mcal) more (*P* = 0.05) ME per day compared with young sows fed 3.18 Mcal/kg ME (20.87 Mcal) per day (Table 5). Young sows fed 3.33 Mcal/kg ME were 6.1% more (*P* < 0.01) more feed efficient than young sows fed 3.18 Mcal/kg ME (Table 5).

### Young sow farrowing performance

There were no interactions (*P* = 0.23) for total pigs born to young sows (Table 6). There tended (*P* = 0.08) to be an interaction for number of pigs born alive to young sows. Young sows fed 1.11% SID Lys and 3.33 Mcal/kg ME tended (*P* ≤ 0.08) to have at least 0.90 fewer pigs born alive compared to young sows fed 0.85% SID Lys and 3.33 Mcal/kg ME and young sows fed 1.11% SID Lys and 3.18 Mcal/kg ME (Table 6). Because of this, there tended (*P* = 0.10) to be an interaction in the percentage of pigs born alive to young sows. Young sows fed 1.11% SID Lys and 3.33 Mcal/kg ME had (*P* = 0.04) 3.48 percentage units fewer pigs born alive compared to young sows fed 1.11% SID Lys and 3.18 Mcal/kg ME. There were no interactions among young sows for the number (*P* = 0.23) or percentage of stillbirths (*P* = 0.11), number (*P* = 0.99) or percentage of mummies (*P* = 0.59), number (*P* = 0.16) or percentage of mortalities that occurred prior to cross fostering (*P* = 0.25), starting litter inventory (*P* = 0.76), or number (*P* = 0.52) or percentage of falloffs (*P* = 0.85, Table 6). The percentage of falloff pigs for young sows fed 3.33 Mcal/kg ME tended (*P* = 0.09) to be 2.37 percentage units less than young sows fed 3.18 Mcal/kg ME (Table 6). There were no interactions (*P* ≥ 0.29) among young sows for the number (*P* = 0.32) or percentage (*P* = 0.29) of mortalities that occurred after cross fostering. However young sows fed 3.18 Mcal/kg ME had 0.31 more (*P* < 0.01) post-cross foster mortalities (2.09 percentage units more) compared to young sows fed 3.33 Mcal/kg ME (Table 6). There were no interactions (*P* = 0.30) among young sows for total removals that occurred after cross fostering. However, young sows fed 3.18 Mcal/kg ME had 0.56 more (*P* ≤ 0.01) post-cross foster removals (4.46 percentage units more) compared to young sows fed 3.33 Mcal/kg ME. There was no interaction for the number of pigs weaned. Young sows fed 0.85% SID Lys weaned 0.60 more (*P* = 0.04) pigs than young sows fed 1.11% SID Lys. Additionally, young sows fed 3.33 Mcal/kg ME tended (*P* = 0.07) to wean 0.54 more pigs than young sows fed 3.18 Mcal/kg ME (Table 6).

**Table 6.** Interactive least squares means of farrowing performance of young sows (parity 1 and 2)^1^

	Treatment (% SID lysine and Mcal/kg, metabolizable energy)					*P* values		
	0.85	0.85	1.11	1.11				Lysine ×
Item	3.18	3.33	3.18	3.33	SEM	Lysine	Energy	Energy
Sows, *n*	99	92	89	91				
Parity	1.51	1.51	1.52	1.53	0.53	0.79	0.88	0.96
Total pigs born, *n*	15.58	15.90	15.74	15.16	0.38	0.45	0.74	0.23
Pigs born alive, *n*	14.69	15.01	15.06	14.11	0.48	0.47	0.38	0.08
Pigs born alive, %	93.83	94.16	94.96	91.48	1.55	0.50	0.17	0.10
Stillbirths, *n*	0.92	0.81	0.68	0.95	0.21	0.76	0.59	0.23
Stillbirths, %	5.17	4.35	3.89	6.38	1.39	0.72	0.42	0.11
Mummies, *n*	0.21	0.32	0.24	0.34	0.07	0.74	0.16	0.99
Mummies, %	1.28	1.76	1.43	2.42	0.47	0.38	0.11	0.59
Pre-cross foster mortalities^2^, *n*	0.65	0.67	0.74	0.50	0.10	0.66	0.26	0.16
Pre-cross foster mortalities^2^, %	4.64	4.67	4.91	3.31	0.73	0.45	0.28	0.25
Starting litter inventory, *n*	15.28	15.15	15.16	14.86	0.40	0.49	0.47	0.76
Falloffs^3^, *n*	0.77	0.64	1.06	0.68	0.20	0.39	0.19	0.52
Falloffs^3^, %	6.15	4.04	7.29	4.65	1.44	0.54	0.09	0.85
Post-cross foster mortalities^4^, *n*	0.73	0.52	0.88	0.46	0.11	0.67	<0.01	0.32
Post-cross foster mortalities^4^, %	4.72	3.40	6.07	3.22	0.74	0.42	<0.01	0.29
Post-cross foster removals^5^, *n*	1.49	1.16	1.93	1.14	0.22	0.34	0.01	0.30
Post-cross foster removals^5^, %	10.87	7.44	13.35	7.87	1.61	0.36	<0.01	0.51
Pigs weaned, *n*	13.51	14.09	12.95	13.45	0.38	0.04	0.07	0.88

^1^A total of 371 parity 1 and 2 sows were used from farrowing until weaning. Sows were weighed, blocked by parity and randomly assigned to 1 of 4 diets with factors being SID lysine (0.85% or 1.11%) and metabolizable energy (3.18 Mcal/kg or 3.33 Mcal/kg). Movement from gestation to farrowing occurred at a target of 112 d of gestation. Weaning occurred at days 14 to 24 of lactation.

^2^Pre-cross foster mortalities—mortalities that occurred before the 48-h cross fostering period.

^3^Falloffs—piglets removed after cross fostering due to weight loss or injury. Those piglets were moved to a nurse sow and removed from the trial.

^4^Post-cross foster mortalities—mortalities that occurred after the 48-h cross fostering period.

^5^Post-cross foster removals—the combination of falloffs and mortalities that occurred after the 48-h cross fostering period.

### Young sow pig performance

There were no interactions between SID Lys and ME among young sows for litter birth weight (*P* = 0.14), litter starting weight (*P* = 0.78), litter weaning weight (*P* = 0.21), litter average daily gain (*P* = 0.64), total litter gain (*P* = 0.38), individual pig birth weight (*P* = 0.57), average pig starting weight (*P* = 0.23), average pig weaning weight (*P* = 0.48), or average pig daily gain (*P* = 0.32, Table 7).

**Table 7.** Interactive least squares means of litter and individual pig performance from young sows (parity 1 and 2)^1^

	Treatment (% SID lysine and Mcal/kg, metabolizable energy)					*P* values		
	0.85	0.85	1.11	1.11				Lysine ×
Item	3.18	3.33	3.18	3.33	SEM	Lysine	Energy	Energy
Litter performance								
Litter birth weight, kg	19.77	19.95	20.21	19.13	0.51	0.76	0.28	0.14
Litter starting weight^2^, kg	20.99	20.94	20.80	21.08	0.76	0.97	0.85	0.78
Litter weaning weight, kg	65.87	68.45	65.19	70.74	1.52	0.49	<0.01	0.21
Litter ADG, kg	2.76	2.89	2.74	2.91	0.06	0.99	<0.01	0.64
Total litter gain^3^, kg	49.14	50.85	48.54	51.95	0.99	0.80	<0.01	0.38
Individual piglet performance								
Average birth weight, kg	1.42	1.40	1.42	1.42	0.03	0.61	0.53	0.57
Average starting weight^2^, kg	1.43	1.42	1.40	1.45	0.03	0.94	0.61	0.23
Average weaning weight, kg	5.07	5.55	5.08	5.17	0.30	0.50	0.31	0.48
Average daily piglet gain, g	15.25	17.31	15.66	14.86	1.55	0.47	0.66	0.32

^1^A total of 371 parity 1 and 2 sows were used from farrowing until weaning. Sows were weighed, blocked by parity and randomly assigned to 1 of 4 diets with factors being SID lysine (0.85% or 1.11%) and metabolizable energy (3.18 Mcal/kg or 3.33 Mcal/kg). Movement from gestation to farrowing occurred at a target of 112 d of gestation. Weaning occurred at days 14 to 24 of lactation.

^2^Litter starting weight = weight of 48-h cross fostering period.

^3^Total litter gain = litter weaning weight − litter starting weight.

Young sows fed 3.33 Mcal/kg ME raised litters were 4.06 kg heavier (*P* < 0.01) at weaning, gained 148 g more (*P* < 0.01) weight per day, and accumulated 2.6 kg more (*P* < 0.01) more total litter weight gain during lactation compared to young sows fed 3.18 Mcal/kg (Table 7).

### Mature sow performance

There were no interactions between SID Lys and ME among mature sows for caliper score into farrowing (*P* = 0.53), caliper score at weaning (*P* = 0.30), body condition score into farrowing (*P* = 0.96), body condition score at weaning (*P* = 0.47), backfat thickness into farrowing (*P* = 0.79), backfat thickness at weaning (*P* = 0.87), change in backfat thickness during lactation (*P* = 0.72), sow weight into farrowing (*P* = 0.11), estimated sow weight after farrowing (*P* = 0.13), conceptus weight (*P* = 0.63), sow weight at weaning (*P* = 0.18), sow weight change during lactation (*P* = 0.76), percentage of sows culled after lactation (*P* = 0.94), or percentage of sows bred by day 4 after weaning (*P* = 0.34, Table 8). There tended to be an interaction for percentage of mature sows bred by day 7 (Figure 2). There were no differences (*P* = 0.21) in the percentage of sows bred by day 7 between sows fed 0.85% SID Lys, but there tended to be an increase (*P* = 0.06) of 7.0 percentage units of mature sows bred by day 7 when fed 1.11% SID Lys and 3.18 Mcal/kg ME compared with mature sows fed 1.11% SID Lys and 3.33 Mcal/kg ME (Figure 2). There was also a tendency for an interaction (*P* = 0.05) for wean-to-estrus interval where there were no differences (*P* = 0.98) between mature sows fed 0.85% SID Lys and 3.18 Mcal/kg ME (5.83 d) and mature sows fed 1.11% SID Lys and 3.33 Mcal/kg ME (5.85 d, Figure 3). There were also no differences (*P* = 0.22) among mature sows fed 0.85% SID Lys regardless of energy. On the other hand, days required to return to estrus between mature sows fed 1.11% SID Lys and 3.33 Mcal/kg ME and mature sows fed 1.11% SID Lys and 3.18 Mcal/kg ME was not statistically different (*P* = 0.11), mature sows fed 1.11% SID Lys and 3.33 Mcal/kg ME had WTE intervals that were 1.47 d longer (Figure 3).

**Table 8.** Interactive least squares means of mature sow (parity 3 to 5) weight and body composition^1^

	Treatment (% SID lysine and Mcal/kg, metabolizable energy)					*P* values		
	0.85	0.85	1.11	1.11				Lysine ×
Item	3.18	3.33	3.18	3.33	SEM	Lysine	Energy	Energy
Sows, *n*	97	84	94	95				
Parity	4.04	3.91	3.99	3.97	0.10	0.97	0.39	0.48
Caliper at entry into farrowing	8.13	7.81	8.42	8.36	0.21	0.04	0.36	0.53
Caliper at weaning	8.40	8.14	7.99	8.15	0.26	0.33	0.83	0.30
Body condition score (BCS) into farrowing	2.83	2.82	2.89	2.88	0.05	0.21	0.92	0.96
Body condition score (BCS) at weaning	2.79	2.77	2.77	2.82	0.06	0.68	0.76	0.47
Backfat at entry into farrowing, mm	10.36	10.04	10.20	10.01	0.25	0.70	0.28	0.79
Backfat at weaning, mm	9.98	9.86	9.36	9.16	0.29	<0.01	0.50	0.87
Backfat change during lactation, mm	-0.29	−0.14	−0.75	−0.77	0.31	0.03	0.78	0.72
Sow weight into farrowing, kg	247.20	242.04	244.05	245.91	2.32	0.87	0.46	0.11
Estimated sow weight after farrowing^2^, kg	219.56	213.61	216.50	216.91	2.14	0.95	0.19	0.13
Conceptus weight^3^, kg	31.38	32.18	31.23	32.59	0.68	0.83	0.06	0.63
Sow weight at weaning, kg	226.13	221.33	221.67	222.90	2.32	0.51	0.42	0.18
Sow weight change during lactation^4^, kg	6.57	7.72	5.67	5.99	1.43	0.34	0.60	0.76
Sow weight change during lactation, %	3.11	3.79	2.78	2.87	0.68	0.33	0.55	0.65
Culled sows, %	14.43	17.86	14.89	18.95	4.07	0.84	0.34	0.94
Sows bred by day 4 after weaning, %	73.08	79.71	79.75	77.03	5.06	0.68	0.69	0.34

^1^A total of 370 parity 3 to 5 sows were used from farrowing until weaning. Sows were weighed, blocked by parity and randomly assigned to 1 of 4 diets with factors being SID lysine (0.85% or 1.11%) and metabolizable energy (3.18 Mcal/kg or 3.33 Mcal/kg). Movement from gestation to farrowing occurred at a target of 112 d of gestation. Weaning occurred at days 14 to 24 of lactation.

^2^Estimated sow weight after farrowing = Adjusted weight before Farrowing − conceptus weight. Adjusted weight before farrowing = total piglets born × 0.039 × days until farrowing from time of last weighing + weight into farrowing (Estrada et al., 2024, doi: 10.1093/jas/skae093). Sow weight into farrowing was the weight of the sow when moved into the farrowing crate at approximately 112 d of gestation.

^3^Conceptus weight = 0.137 + 1.329 × total piglets born × average piglet birth weight (Estrada et al., 2024, doi: 10.1093/jas/skae093).

^4^Sow weight change = weight at weaning − estimated sow weight after farrowing.

Mature sows fed 1.11% SID Lys had caliper measurements at entry into farrowing that were 0.42 units greater (*P* = 0.04) than mature sows fed 0.85 % SID Lys. Contrarily, there were no differences (*P* = 0.70) backfat thickness going into farrowing of mature sows fed 0.85% SID Lys (10.2 mm) and mature sows fed 1.11% SID Lys (10.1 mm, Table 8). On the other hand, backfat thickness at weaning of mature sows fed 1.11% SID Lys was 0.66 mm less than (*P* < 0.01) mature sows fed 0.85% SID Lys. Furthermore, mature sows fed 1.11% SID Lys experienced a greater (*P* = 0.03) decrease in backfat thickness during lactation compared with mature sows fed 0.85% SID Lys (Table 8). Mature sows fed 3.33 Mcal/kg ME tended (*P* = 0.06) to have conceptus weights that were 1.08 kg greater than mature sows fed 3.18 Mcal/kg ME (Table 8).

### Mature sow feed consumption

There were no interactions between SID Lys and ME among mature sows for daily g/d SID Lys intake (*P* = 0.30), Mcal/d daily ME intake (*P* = 0.35), loading to weaning feed intake (*P* = 0.57), lactation total intake (*P* = 0.43), lactation daily feed intake (*P* = 0.39), or lactation gain-to-feed ratio (*P* = 0.92, Table 9).

**Table 9.** Interactive least squares means of total feed intake and average daily feed intake of mature sows (parity 3 - 5) during lactation^1^

	Treatment (% SID lysine and Mcal/kg, metabolizable energy)					*P* values		
	0.85	0.85	1.11	1.11				Lysine ×
Item	3.18	3.33	3.18	3.33	SEM	Lysine	Energy	Energy
Sows, *n*	97	84	94	95				
Parity	4.04	3.91	3.98	3.97	0.10	0.97	0.37	0.48
Daily SID lysine intake, g/d	65.10	64.54	82.18	79.38	1.10	<0.01	0.11	0.30
Daily metabolizable energy intake, Mcal/d	24.37	25.28	23.65	23.92	0.36	<0.01	0.09	0.35
Loading to weaning total intake^2^, kg	147.15	146.20	141.72	138.16	2.92	<0.01	0.32	0.57
Loading to weaning ADFI^2^, kg	6.51	6.48	6.32	6.16	0.11	<0.01	0.33	0.49
Lactation total intake, kg	135.86	135.67	131.97	128.08	3.03	0.02	0.40	0.43
Lactation ADFI, kg	7.66	7.59	7.44	7.18	0.11	<0.01	0.13	0.39
Lactation G:F^3^	0.34	0.35	0.37	0.38	<0.01	<0.01	0.28	0.92

^1^A total of 370 parity 3 to 5 sows were used from farrowing until weaning. Sows were weighed, blocked by parity and randomly assigned to 1 of 4 diets with factors being SID lysine (0.85% or 1.11%) and metabolizable energy (3.18 Mcal/kg or 3.33 Mcal/kg). Movement from gestation to farrowing occurred at a target of 112 d of gestation. Weaning occurred at days 14 to 24 of lactation.

^2^Time of movement from gestation to farrowing room through weaning of the piglets.

^3^Lactation G:F = (litter weaning weight − litter starting weight) ÷ sow total lactation feed intake.

Mature sows fed 1.11% SID Lys (80.78 g) consumed 19.7% (15.93 g) more (*P* < 0.01) SID Lys per day compared with mature sows fed 0.85% SID Lys (64.85 g; Table 9). Mature sows fed 0.85% SID Lys (24.83 Mcal/d) consumed 4.2% (1.04 Mcal) more (*P* < 0.01) ME per day compared with mature sows fed 1.11% SID Lys (23.79 Mcal/d) per day (Table 9). Mature sows fed 0.85% SID Lys consumed 5.74 kg more (*P* = 0.02) feed during lactation compared with mature sows fed 1.11% SID Lys (Table 9). Mature sows fed 1.11% SID Lys were 6.9% more (*P* < 0.01) feed efficient during lactation compared with mature sows fed 0.85% SID Lys (Table 9).

Mature sows fed 3.33 Mcal/kg ME (24.60 Mcal) tended (*P* = 0.09) to consume 2.4% (0.59 Mcal) more ME per day compared with young sows fed 3.18 Mcal/kg ME (24.01 Mcal) per day (Table 9). There was no difference (*P* = 0.40) in total lactation feed intake or daily feed intake (*P* = 0.13) between mature sows fed 3.18 Mcal/kg ME (133.92 kg) and mature sows fed 3.33 Mcal/kg ME (131.87 kg, Table 9). There were also no differences in lactation gain-to-feed ratio between mature sows fed 3.18 Mcal/kg ME (0.36) and mature sows fed 3.33 Mcal/kg ME (0.37, Table 9).

### Mature sow farrowing performance

There were no interactions between SID Lys and ME among mature sows for total pigs born (*P* = 0.99), number of pigs born alive (*P* = 0.81), percentage of pigs born alive (*P* = 0.81), stillbirths (*P* = 0.91), percentage of stillbirths (*P* = 0.89), number of mummies (*P* = 0.47), percentage of mummies (*P* = 0.40), pre-cross foster mortalities (*P* = 0.15), number of falloff pigs (*P* = 0.14), percentage of fall off pigs (*P* = 0.13), post-cross foster removal (*P* = 0.97), or percentage of cross foster removals (*P* = 0.98) or pigs weaned (*P* = 0.47, Table 10).

**Table 10.** Interactive least squares means of farrowing performance of mature sows (parity 3 - 5)^1^

	Treatment (% SID lysine and Mcal/kg, metabolizable energy)					*P* values		
	0.85	0.85	1.11	1.11				Lysine ×
Item	3.18	3.33	3.18	3.33	SEM	Lysine	Energy	Energy
Sows, *n*	97	84	94	95				
Parity	4.04	3.95	4.00	4.01	0.10	0.93	0.63	0.53
Total pigs born, *n*	17.28	17.90	17.44	18.06	0.40	0.67	0.11	0.99
Pigs born alive, *n*	15.61	15.90	15.70	15.93	0.44	0.88	0.48	0.93
Pigs born alive, %	91.06	90.95	89.96	90.39	1.43	0.47	0.89	0.81
Stillbirths, *n*	1.46	1.53	1.67	1.70	0.21	0.35	0.80	0.91
Stillbirths, %	8.35	8.16	8.89	9.00	1.08	0.51	0.97	0.89
Mummies, *n*	0.21	0.26	0.28	0.20	0.11	0.94	0.86	0.47
Mummies, %	1.03	1.32	1.60	1.06	0.63	0.75	0.79	0.40
Pre-cross foster mortalities^2^, *n*	0.82	1.09	1.09	0.97	0.14	0.59	0.59	0.15
Pre-cross foster mortalities^2^, %	5.43	6.85	7.49	6.27	1.01	0.40	0.91	0.13
Starting litter inventory, *n*	14.80	14.59	14.76	14.88	0.19	0.40	0.75	0.26
Falloffs^3^, *n*	1.09	0.91	0.88	1.13	0.16	0.99	0.81	0.14
Falloffs^3^, %	7.53	6.21	5.95	7.58	1.03	0.92	0.87	0.13
Post-cross foster mortalities^4^, *n*	0.91^ab^	1.15^b^	1.00^ab^	0.82^a^	0.13	0.24	0.78	0.04
Post-cross foster mortalities^4^, %	6.24^ab^	7.96^b^	6.91^ab^	5.60^a^	0.92	0.26	0.78	0.04
Post-cross foster removals^5^, *n*	2.08	2.15	1.96	2.03	0.24	0.53	0.74	0.97
Post-cross foster removals^5^, %	14.18	14.58	13.29	13.61	1.64	0.48	0.78	0.98
Pigs weaned, *n*	12.62	12.35	12.70	12.76	0.23	0.28	0.63	0.47

^1^A total of 370 parity 3 to 5 sows were used from farrowing until weaning. Sows were weighed, blocked by parity and randomly assigned to 1 of 4 diets with factors being SID lysine (0.85% or 1.11%) and metabolizable energy (3.18 Mcal/kg or 3.33 Mcal/kg). Movement from gestation to farrowing occurred at a target of 112 d of gestation. Weaning occurred at days 14 to 24 of lactation.

^2^Pre-cross foster mortalities—mortalities that occurred before the 48-h cross fostering period.

^3^Falloffs—piglets removed after cross fostering due to weight loss or injury. Those piglets were moved to a nurse sow and removed from the trial.

^4^Post-cross foster mortalities—mortalities that occurred after the 48-h cross fostering period.

^5^Post-cross foster removals—the combination of falloffs and mortalities that occurred after the 48-h cross fostering period.

There was an interaction (*P* = 0.04) for post-cross foster mortalities where there were no differences in the number of pig mortalities for mature sows fed 3.18 Mcal/kg ME when SID Lys levels were different, but post-cross foster pig mortalities of mature sows fed 3.33 Mcal/kg ME was less when sows 1.11% SID Lys compared with mature sows fed 0.85% SID Lys (Table 10).

There were no main effect differences (*P* ≥ 0.24) between mature sows fed 0.85% SID Lys and mature sows fed 1.11% SID Lys (Table 10). There were also no main effect differences between mature sows fed 3.18 Mcal/kg ME and mature sows fed 3.33 Mcal/kg ME for total pigs born (*P* = 0.11), percentage of pigs born alive (*P* = 0.89), number of stillbirths (*P* = 0.80), percentage of stillbirths (*P* = 0.97), number of mummies (*P* = 0.86), percentage of mummies (*P* = 0.79), pre-cross foster mortalities (*P* = 0.59), starting litter inventory (*P* = 0.75), number of falloff pigs (*P* = 0.81), percentage of falloff pigs (*P* = 0.87), post-cross foster mortalities (*P* = 0.78), or the number of pigs weaned (*P* = 0.63) (Table 10). Mature sows fed 3.33 Mcal/kg ME tended (*P* = 0.10) to have 0.58 more pigs born alive compared with mature sows fed 3.18 Mcal/kg ME (Table 10).

### Mature sow pig performance

There were no interactions between SID Lys and ME among mature sows for litter birth weight (*P* = 0.54), litter starting weight (*P* = 0.34), litter weaning weight (*P* = 0.66), total litter gain (*P* = 0.81), or average individual pig birth weight (*P* = 0.99, Table 11).

**Table 11.** Interactive least squares means of litter and individual pig performance from mature sows (parity 3 to 5)^1^

	Treatment (% SID lysine and Mcal/kg, metabolizable energy)					*P* values		
	0.85	0.85	1.11	1.11				Lysine ×
Item	3.18	3.33	3.18	3.33	SEM	Lysine	Energy	Energy
Litter performance								
Litter birth weight, kg	21.75	22.24	21.35	22.37	0.52	0.76	0.08	0.54
Litter starting weight^2^, kg	20.86	21.02	21.71	21.03	0.51	0.33	0.55	0.34
Litter weaning weight, kg	64.69	65.38	67.13	66.89	1.79	0.20	0.96	0.66
Litter ADG, kg	2.80	2.81	2.88	2.87	0.06	0.22	0.99	0.77
Total litter gain^3^, kg	46.68	47.43	48.25	48.50	1.40	0.23	0.65	0.81
Individual piglet performance								
Average birth weight, kg	1.42	1.41	1.42	1.40	0.03	0.88	0.51	0.99
Average starting weight^2^, kg	1.43	1.47	1.50	1.44	0.03	0.51	0.76	0.06
Average weaning weight, kg	5.05^a^	5.21^b^	5.22^b^	5.10^ab^	0.09	0.68	0.73	0.04
Average daily piglet gain, g	16.98	17.71	17.28	16.61	0.43	0.28	0.94	0.06

^1^A total of 370 parity 3 to 5 sows were used from farrowing until weaning. Sows were weighed, blocked by parity and randomly assigned to 1 of 4 diets with factors being SID lysine (0.85% or 1.11%) and metabolizable energy (3.18 Mcal/kg or 3.33 Mcal/kg). Movement from gestation to farrowing occurred at a target of 112 d of gestation. Weaning occurred at dats 14 to 24 of lactation.

^2^Litter starting weight = weight of 48-h cross fostering period.

^3^Total litter gain = litter weaning weight − litter starting weight.

Mature sows fed0.85% SID Lys and 3.33 Mcal/kg ME and mature sows fed 1.11% SID Lys and 3.18 Mcal/kg ME weaned pigs that were at least 187 g heavier (*P* ≤ 0.05) than mature sows fed 0.85% SID Lys and 3.18 Mcal/kg ME (Table 11). In other words, increasing either SID Lys or ME increased individual pig weaning weight. However, there were no differences (*P* ≥ 0.26) in individual pig weaning weight among sows fed 0.85% SID Lys and 3.33 Mcal/kg, those fed 1.11% SID Lys and 3.18 Mcal/kg, and those fed 1.11% SID Lys and 3.33 Mcal/kg ME (Table 11). This means increasing both SID Lys and ME provided no advantage in pig weaning weight compared to just increasing one of the 2 nutrients.

Pigs from mature sows fed 1.11% SID Lys and 3.18 Mcal/kg ME had average starting weights that tended (*P* = 0.06) to be 0.07 kg greater than the pigs from mature sows fed 0.85% SID Lys and 3.18 Mcal/kg ME. There also tended (*P* = 0.06) to be an interaction for average daily piglet gain (Table 11). Pigs born to mature sows fed 1.11% SID Lys and 3.33 Mcal/kg ME gained 1.09 kg less than (*P* = 0.04) pigs born to mature sows fed 0.85% SID Lys and 3.33 Mcal/kg ME (Table 11).

## Discussion

Adequate Lys and energy intakes are necessary for the success of today’s high-producing sows. Several historical studies have evaluated the Lys requirements of lactating sows (Lewis and Speer, 1973; Kim et al., 1999; Yang et al., 2000) and the Lys and energy requirements of gestating sows (Cooper et al., 2001; Gourley et al., 2020).

Estrada et al. (2024) reported that gilts and sows (parity 1 to 8) that consumed 53.49 g/d SID Lys lost 3.47% of their body weight during lactation compared with sows that consumed 78.77 g/d SID Lys which increased body weight by 1.43%. Other previous studies reported that greater Lys and ME intake minimizes body weight loss of lactating sows (Doumad et al., 1998; Kusina et al., 1999; Xue et al., 2012). However, in the current study, there were no differences in body weights of young or mature sows after farrowing (accounting for the loss in weight from the pigs and conceptus during farrowing) or at weaning. Yang et al. (2008) reported a 13.8% increase in backfat thickness in sows when SID Lys intake increased from 48.9 g/d SID Lys to 50.2 g/d SID Lys and ME intake increased 6.8% in diets formulated to include 1.04% SID Lys. Backfat increased by 23.3% when SID Lys was approximately 65 g SID Lys/d in diets that included 1.3% SID Lys, and ME intake increased 4.2% (Yang et al., 2008).

There were no differences in the change in backfat thickness during lactation for young sows in the current trial regardless of SID Lys treatment (*P* = 0.87) or ME treatment (*P *= 0.81). That was not the case for mature sows. Sows fed 1.11% SID Lys lost 0.68 mm in backfat thickness during lactation, but sows fed 0.85% SID Lys only lost 0.20 mm of backfat thickness during lactation. The differences in body composition may be attributed to total feed intake. Mature sows fed 0.85% SID Lys consumed 316 g more (*P* < 0.01) feed each day of lactation compared with sows fed 1.11% SID Lys. This resulted in a cumulative difference in total feed intake of 5.70 kg or an increase of 4.3% during lactation. Yang et al. (2000) reported ADFI decreased by 300 g/d during lactation when dietary lysine concentration increased from 0.85% to 1.10% in first and second parity sows and by 100 g/d in third parity sows. Several studies reported that feeding high dietary energy reduced feed intake (Pettigrew and Moser, 1991; Xue et al., 2012). Xue et al. (2012) observed feed intake was decreased by 220 g/d in first parity sows and by 580 g/d in parity 3 + sows when fed 3.40 Mcal/kg ME compared to 3.20 Mcal/kg ME.

Recently Tokach et al. (2019) estimated the SID requirement for a lactating sow was approximately 27 g/d per kg of litter growth. Based on the growth rate of litters in this trial (approximately 2.6 kg/d), sows would need to consume over 70 g SID Lys/d. Only young and mature sows fed 1.11% SID Lys had daily Lys intakes at that level. Diets were formulated in this trial to deliver 62 or 80 g SID Lys per day to sows that consume 7.3 kg of complete feed per day, spanning the estimated SID Lys needs described by Tokach et al. (2019). Overall, sows in this trial consumed 61g SID Lys per day or 76 g SID Lys per day depending on their treatment assignments. That means the population average consumed excess Lys relative to the estimated requirements. Still, sows fed diets containing 0.85% SID Lys consumed 1.6% less lysine per day than targeted for those intended to consume 62 g and sows fed diets containing 1.11% SID Lys consumed 5% less lysine per day than targeted for sows intended to consume 80 g of lysine per day. The same was true for ME intake. Sows fed diets containing 3.18 Mcal/kg ME were intended to consume 23.2 Mcal/d, but instead only consumed 22.6. Meaning they were 2.6% under target. Sows fed diets containing 3.33 Mcal/kg ME were intended to consume 24.3 Mcal/kg but only consumed 23.4 Mcal/d. This resulted in ME consumption being 3.8% less than formulated. This was directly contributed to ADFI differences between young and mature sows. Young sows under consumed targets for SID Lys per day and ME per day wereras mature sows over consumed targets for SID Lys per day.

Regardless of differences in Lys consumption, there were few differences in pig performance between young or mature sows fed 0.85% SID Lys or 1.11% SID Lys. Sow diets are typically formulated to meet the needs of the herd average daily feed intake, but it is well established that older parity sows consume more feed than younger sows that are still growing (Estrada et al., 2024). Recent studies evaluating the optimum synergistic Lys and ME levels during lactation by parity are scarce. However, the differences in nutritional demands of young and old sows must be considered when formulating lactation diets. Estrada et al. (2024) reported that ADFI increased by 22.82% from parity 1 to parity 2, and by 9.36% from parity 2 to parity 3+. These differences in ADFI among parity groups resulted in young sows (e.g., average of P1 and P2) consuming 62 g/d SID Lys and up to a 30% variation in SID Lys intake among the population (Estrada et al., 2024). Increasing SID Lys intake by 20.5% to the level of SID Lys intake of mature sows (i.e., P3+), increased individual pig weaning weight by 8.4% (0.47 kg, Estrada et al., 2024).

In a similar experiment, sows that consumed 54 g SID Lys/d were able to wean pigs that gained 0.23 kg/d (Xue et al., 2012) which is at least 20 g more per day than pigs in the current trial. It should be noted sows in Xue et al. (2012) weaned less than 10 pigs per litter, and sows in this trial weaned more than 12 pigs. Still further, Hojgaard et al. (2019) reported a linear increase in litter daily gain as sows SID Lys intake increased from 39.4 g/d to 63.9 g/d, but there were no differences in litter daily gain between sows that consumed between 53.5 g/d and sows that consumed 63.9 g/d. Based on these examples it should be no surprise that litter daily gain was not improved when sows consumed greater daily SID Lys intakes, even in young sows that only consumed 55.88 g SID Lys per day.

Overall, increasing lysine fed to sows in this trial had no impact on young sow farrowing performance but increasing ME reduced mortality and morbidity of pigs born to young sows. Differences in starting inventories between young sows fed 3.33 Mcal/kg and young sows fed 3.18 Mcal/kg was 0.22 pigs per litter (*P* = 0.09). Differences in pigs weaned between young sows fed 3.33 Mcal/kg and young sows fed 3.18 Mcal/kg was 0.66 pigs per litter (*P* < 0.01).

That means only 33% of the difference in weaned weight was due to differences in starting inventories. Or, 66% of the difference was due to greater mortality rates of pigs from young sows fed 3.18 Mcla/kg. There was a 6.2% increase in litter weaning weight of litters from young sows fed 3.33 Mcal/kg ME compared with young sows fed 3.18 Mcal/kg ME, but no differences in individual pig weaning weights. This is largely the result of a 0.45 percentage unit increase in mortality rate of litters from sows fed 3.18 Mcal/kg ME compared with litters from young sows fed 3.33 Mcal/kg ME. The greater mortality rate resulted in a difference in the number of pigs weaned by 0.66 pigs per litter. So, it appears increasing ME in young sow lactation diets does not improve growth performance of individual pigs, but it does reduce litter mortality resulting in a net litter performance improvement. Further, the reduction in mortality and increase in the number of pigs weaned resulted in a 7.9% increase in lactation feed efficiency of young sows fed 3.33 Mcal/kg ME compared with young sows fed 3.18 Mcal/kg. Again, this is likely a more of a result of greater ME intake reducing mortality than it is an improvement in individual pig performance. Previously, litter size did not influence lactation feed intake of sows as pigs weaned increased from 7 to 15 pigs (Eissen et al., 2000). Logically then, nursing larger litters mathematically improves feed efficiency as litter weight inherently increases as pig numbers increase. Conversely, there were no differences in lactation feed efficacy between mature sows fed 3.18 Mcal/kg ME and mature sows fed 3.33 Mcal/kg ME. This actually aligns with the response to energy in young sows. In mature sows there were no differences in starting inventory (*P* = 0.75), pre-weaned mortality (*P* = 0.78), or pigs weaned per litter (*P* = 0.63). Ultimately, increasing energy to young sows from 3.18 to 3.33 Mcal/kg ME increased litter weaning weight, increased litter gain during lactation, and reduced pig removals, Litter performance in mature sows was unaffected by ME level. Gourley et al. (2020) theorized that the difference in response to increasing energy between young and mature sows may be due to older sows having a lesser requirement for ME where additional energy fed to mature sows may not be partitioned to litter growth. This is different from young sows where they are likely growing themselves. Similar to young sows, there were no differences in individual pig performance when ME was increased in mature sows. Further confirming improvements in litter performance by increasing ME in young sow lactations diets was actually due to a reduction in mortality and not differences in individual pig performance. Unlike in young sows, increasing either SID Lys or ME increased individual pig weaning weight. However, there were no differences (*P* ≥ 0.26) in individual pig weaning weight among sows fed 0.85% SID Lys and 3.33 Mcal/kg, those fed 1.11% SID Lys and 3.18 Mcal/kg, and those fed 1.11% SID Lys and 3.33 Mcal/kg ME (Table 11). This means increasing both SID Lys and ME provided no advantage in pig weaning weight compared to just increasing one of the 2 nutrients.

There was a 7.6% improvement (*P* < 0.01) in lactation feed efficiency of mature sows fed 1.11% SID Lys compared with mature sows fed 0.85% SID Lys. Again, different from young sows, increasing SID Lys, ME, or both increased individual pig weaning weight. Interestingly, although not statistically compared, young sows fed 0.85% SID Lys and 3.18 Mcal/kg ME (5.07 kg) weaned individual pigs with similar weights to mature sows fed 0.85% SID Lys and 3.18 Mcal/kg ME (5.05 kg). However, when ME was increased from 3.18 Mcal/kg to 3.33 Mcal/kg in sows fed 3.18% SID Lys, weaning weights of individual pigs in young sow litters increased in weight by 9.5% but only increased by 3.2% in mature sow litters.

Wean-to-estrus interval is a significant factor that affects the reproductive productivity of sows. A short WEI is important for maximizing the number of pigs a sow can produce in a year. In the current study, young sows fed 1.11% SID Lys and 3.33 Mcal/kg ME had a WEI that was more than 4 d longer than young sows fed 1.11% SID Lys and 3.18 Mcal/kg ME, and almost than 3 d longer than young sows fed either of the 0.85% SID Lys treatments. Extending WEI directly reduces pigs weaned per sow per year and negatively impacts profitability. Further, the proportion of young sows bred by day 7 in the 1.11% SID Lys, 3.33 Mcal/kg ME group was at least 14.8 percentage units less than young sows in the other 3 treatment groups. Mature sows fed either 1.11% SID Lys and 3.33 Mcal/kg ME or 0.85% SID Lys and 3.18 Mcal/kg ME had a WEI that was more than 1 d longer than mature sows fed the other 2 treatments. Furthermore, the percentage of mature sows bred by day 7 in the 1.11% SID Lys and 3.33 Mcal/kg ME or the 0.85% SID Lys and 3.18 Mcal/kg ME groups was at least 4.8 percentage units less than mature sows in the other 2 treatment groups.

Based on previous studies, young and mature sows in the current study fed 1.11% SID Lys and 3.33 Mcal/kg ME were expected to have improved or unchanged WEI and the proportion of sows bred by day 7 was expected to increase. In fact, the opposite was true. Feeding young sows an increased level of SID Lys in conjunction with an increased level of energy was detrimental to their subsequent reproductive performance. The same was true for mature sows, though to a lesser magnitude. Reproductive performance of mature sows was improved when either SID Lys or ME was increased, but when both were increased, reproductive performance was compromised. Reproductive performance was also compromised when neither SID Lys nor ME were increased. Increasing both SID Lys and ME decreases subsequent breeding performance by greater magnitudes than increasing SID Lys and ME in mature sows, but reproductive performance is reduced in both sets of sows. Even so, reproductive performance of mature sows appeared to benefit from increasing either SID Lys or ME.

Production strategies must be considered when evaluating dietary lactation lysine and energy inclusion levels. Nutrient utilization reported by Feyera et al. (2020) was better than that of the sows in the current study, perhaps because of less lysine but more likely due to a lactation length that was about 8 d longer than the sows in the current study. The balance between improving lactation feed efficiency with potentially compromised subsequent reproductive performance must be jointly considered. The parity structure of the farm must also be considered as decisions are made for nutrient inclusion levels of lactating sows.

In summary, increasing dietary SID Lys concentrations from 0.85% to 1.11% had little impact on net litter performance of either young or mature sows. This may be a result of all sows regardless of SID Lys treatment consuming adequate Lys levels. On the other hand, feeding 3.33 Mcal/kg ME to young sows improved litter wean weight and litter gain, decreased post-cross foster removals, and improved lactation feed conversion compared to feeding 3.18 Mcal/kg ME. These improvements seemed to be more of a function of decreasing mortality rate of the litter than it did on improving performance of the individual pig but supports the hypothesis that increasing ME in young sows may be necessary for sow development and net productivity of the litter. Feeding 1.11% SID Lys to mature sows improved lactation feed conversion compared to feeding 0.85% SID Lys. However, feeding 3.33 Mcal/kg ME concurrently with 1.11% SID Lys decreased the proportion of young and mature sows that were bred by day 7 and lengthened WEI. In this trial, WEI interval was extended by over 4 d in young sows and nearly 1.5 d in mature sows when fed 1.11% SID Lys and 3.33 Mcal/kg ME compared with other diet combinations. This directly reduces pigs weaned per sow per year by a full pig in young sows and nearly 0.4 of a pig in mature sows. Reproductive performance of mature sows was also negatively affected when fed diets were formulated to deliver more than recommended lysine and energy or low in both lysine and energy. When formulating diets for lactating sows, the differences in nutritional requirements between young and mature sows, as well as the relationship between increased levels of lysine and energy must be considered.

## Abbreviations

AA: amino acid
ADFI: average daily feed intake
ADG: average daily gain
G:F: gain-to-feed
Lys: lysine
ME: metabolizable energy
SID: standard ileal digestible
WEI: wean-to-estrus interval
